# Evaluation of pathological response to neoadjuvant chemotherapy in locally advanced cervical cancer

**DOI:** 10.1186/s12967-024-05482-3

**Published:** 2024-07-14

**Authors:** Li-Jun Wei, Jia Fu, Hai-Xia Yang, Xia Yang, Hao-Yu Liang, Rong-Zhen Luo, Li-Li Liu

**Affiliations:** 1 State Key Laboratory of Oncology in South China, Collaborative Innovation Center for Cancer Medicine, Sun Yat-sen University Cancer Center, Guangzhou, 510060 China; 2 Department of Pathology, Sun Yat-sen University Cancer Center, 651# Dong Feng Road East, Guangzhou, 510060 Guangdong China; 3 Department of Pathology, The Second Affiliated Hospital of Shenzhen University, Shenzhen, 518101 China

**Keywords:** Neoadjuvant chemotherapy, Pathological response, Stromal type, Locally advanced cervical cancer, Prognosis

## Abstract

Neoadjuvant chemotherapy (NACT) is a viable therapeutic option for women diagnosed locally advanced cervical cancer (LACC). However, the factors influencing pathological response are still controversial. We collected pair specimens of 185 LACC patients before and after receiving NACT and conducted histological evaluation. 8 fresh tissues pre-treatment were selected from the entire cohort to conducted immune gene expression profiling. A novel pathological grading system was established by comprehensively assessing the percentages of viable tumor, inflammatory stroma, fibrotic stroma, and necrosis in the tumor bed. Then, 185 patients were categorized into either the good pathological response (GPR) group or the poor pathological response (PPR) group post-NACT, with 134 patients (72.4%, 134/185) achieving GPR. Increasing tumor-infiltrating lymphocytes (TILs) and tumor-infiltrating lymphocytes volume (TILV) pre-treatment were correlated with GPR, with TILV emerging as an independent predictive factor for GPR. Additionally, CIBERSORT analysis revealed noteworthy differences in the expression of immune makers between cPR and non-cPR group. Furthermore, a significantly heightened density of CD8 + T cells and a reduced density of FOXP3 + T cells were observed in GPR than PPR. Importantly, patients exhibiting GPR or inflammatory type demonstrated improved overall survival and disease-free survival. Notably, stromal type was an independent prognostic factor in multivariate analysis. Our study indicates the elevated TILV in pre-treatment specimens may predict a favorable response to NACT, while identifying stromal type in post-treatment specimens as an independent prognostic factor. Moreover, we proposed this pathological grading system in NACT patients, which may offer a more comprehensive understanding of treatment response and prognosis.

**Supplementary Information:**

The online version contains supplementary material available at 10.1186/s12967-024-05482-3.

## Introduction

Cervical cancer is the second most prevalent contributor to both cancer incidence and mortality for women in developing countries [1]. Concurrent chemoradiotherapy (CCRT) is recommended as the treatment for Locally advanced cervical cancer (LACC) [2, 3]. Nevertheless, constraints exist in utilizing CCRT for LACC treatment [4]. Certain studies have reported the utilization of hysterectomy following neoadjuvant chemotherapy (NACT) for LACC, which may reduce tumor sizes, decrease the probability of tumor metastasis and recurrence, rectify pelvic anatomy distortion, ultimately facilitating the enhanced delivery of subsequent therapies [5]. Studies indicate a favorable correlation between superior clinical and pathologic responses to NACT and improved survival rates among LACC patients [6, 7]. Conversely, stable disease post-NACT has also been identified as a harbinger of poor prognosis [8]. Despite NACT being employed globally, consensus guidelines for the optimal patient selection remain elusive.

Previous studies highlight the impact of immune cells in the tumor microenvironment on the efficacy of anticancer therapies [9, 10]. Elevated pre-treatment densities of tumor infiltrating lymphocytes (TILs) correlate positively with complete pathological response (cPR) rates [11]. D’Alessandris et al. observed a significant correlation between the percentage of TILs and PD-L1 expression on pathological response to NACT, indicating the robust immunogenic potential of cervical cancer [10]. Existing studies predominantly focused on TILs percentage, but do not take into account the stroma, which may not fully reflect tumor immunity. We found that tumor infiltrating lymphocytes volume (TILV) has demonstrated superior predictive value for cPR and overall survival in triple-negative invasive breast cancer [12]. TILV was calculated by using the formula TILV = stroma in tumor (%) × stromal TILs (%), which considered both the percentage of TILs and tumor stroma. However, the predictive and prognostic significance of TILV in cervical cancer remains unexplored.

The histopathologic evaluation of surgical specimens represents an ideal for assessing NACT effectiveness. Although the histopathologic assessment of NACT response is established for various solid tumors (e.g., breast, esophagus and ovarian) [13–15], no universally accepted system exists for cervical cancer. Several studies have quantified residual tumors and chemotherapy-induced regressive changes in other tumors, correlating these with patient outcomes [16, 17]. In cervical cancer, previous studies have proposed some pathological response grading method and morphological changes after neoadjuvant therapy [18, 19]. However, they have not been associated with prognosis, and none have achieved widespread adoption in routine clinical practice due to differing criterion. This study aimed to establish a simple, prognostically significant, and reproducible grading system of evaluating LACC patients’ responses to NACT, based on the examination of resection specimens, and can be incorporated into routine histopathology reporting.

## Materials and methods

### Patients and samples

**Fig. 1:**
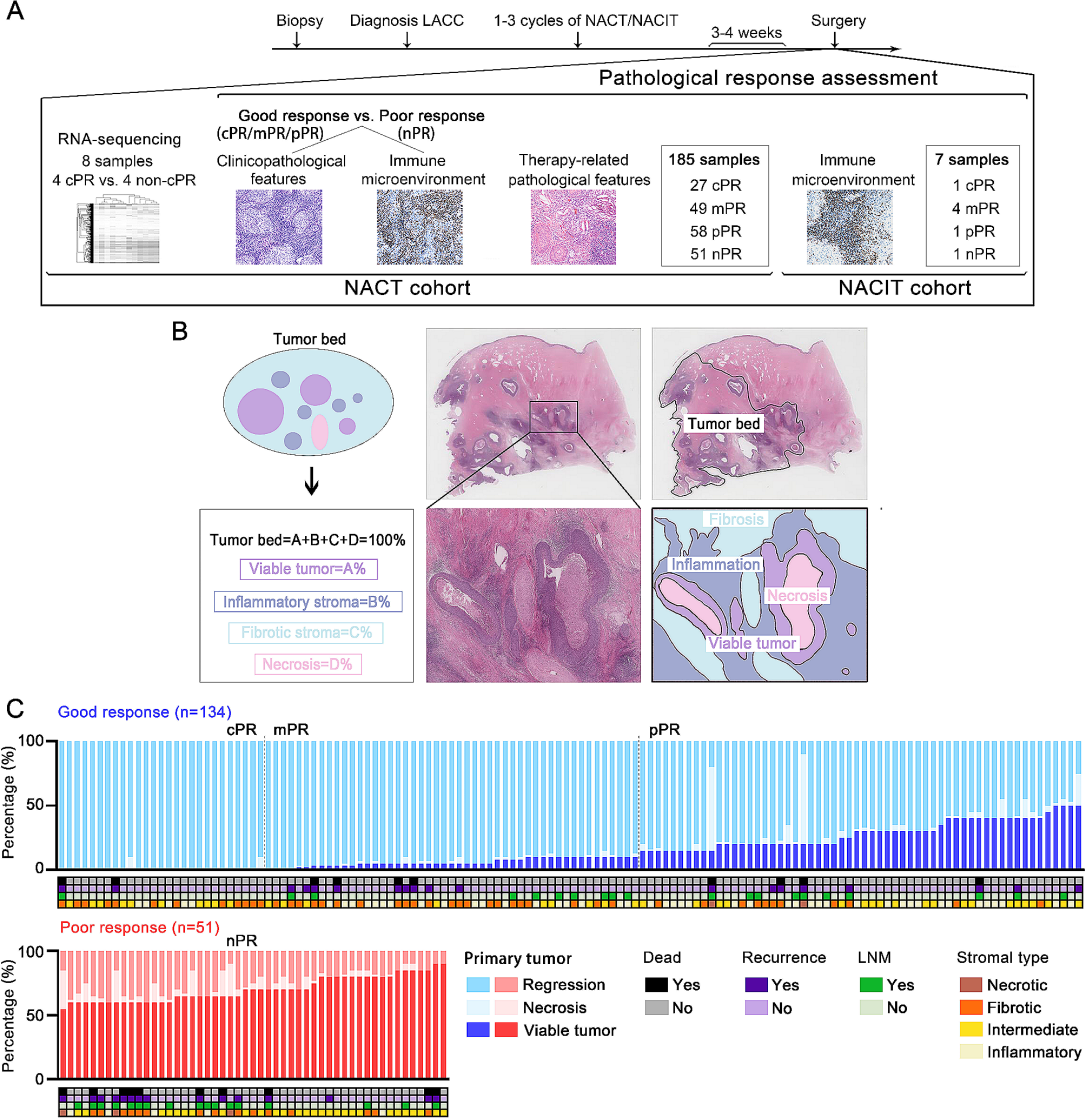
**(A)** Schematic overview of the study design. **(B)** Histological components of tumor bed. **(C)** Evaluation of viable tumor, regression and necrosis in primary tumor and clinicopathological features in 185 LACC patients. NACT, neoadjuvant chemotherapy; NACIT, neoadjuvant chemotherapy combined with immunotherapy; cPR, complete pathological response; mPR, major pathological response; pPR, partial pathological response; nPR, no pathological response; LNM, Lymph node metastatic

The studied cohort were retrospectively retrieved from the pathologic files of Sun Yat-sen University Cancer Center (SYSUCC) between August 1, 2007 and January 31, 2019. Inclusion criteria are as follows: (1) diagnosis of LACC (2018 FIGO stage IB3, IIA2-IVA) from biopsy, including squamous cell carcinomas, adenocarcinomas, adenosquamous carcinoma and neuroendocrine small cell carcinomas. (2) received 1–3 cycles of NACT or neoadjuvant chemotherapy combined with immunotherapy (NACIT). (3) underwent a radical hysterectomy after 3–4 weeks of NACT. Low-grade or high-grade squamous intraepithelial lesion, adenocarcinoma in situ, mesenchymal tumors and germ cell tumors were excluded. Pelvic computed tomography (CT) was performed before NACT and 7–10 days after the last chemotherapeutic course, and the response evaluation criteria in solid tumors (RECIST, v1.1) were applied to score the chemotherapeutic response [20]. Stable disease (SD) refers to < 30% decrease or > 20% increase in the longest diameter; partial response (PR) refers to > 30% decrease in the longest diameter; complete response (CR) refers to tumor complete resolution. Pair specimens of 185 LACC patients before and after receiving NACT were collected, while 7 pair samples before and after receiving NACIT. Both biopsy and resection specimens are available for review on hematoxylin-eosin (H&E) slides. The schematic overview of the study design was shown in Fig. 1A.

### Pathological response evaluation

Pathological response after NACT were evaluated on H&E-stained slides of resection specimens by two experienced pathologists. As shown in Fig. 1B, tumor beds refer to the area where the original tumor was believed to be located before treatment, consists of viable tumor, necrosis, stroma including inflammation and fibrosis, and the total of 4 components is 100% [16]. The percentage of every component was made in 5% or 10% increments as continuous variables, unless the amount less than 10%. In this case, an estimate of single-digit percentages should be recorded. Every slide that tumor bed can be recognized was reviewed, as well as each proportion of 4 components was given and then average was taken respectively. As proposed in previous studies [21], pathological responses were classified into 4 categories according to the proportion of residual viable tumor: complete pathological response (cPR) was defined as the absence of viable tumor; major pathological response (mPR) refers to viable tumor > 0 but ≤ 10%; partial pathological response (pPR) refers to viable tumor > 10% but ≤ 50%; no pathological response (nPR) refers to viable tumor > 50%. Patients who obtained cPR, mPR and pPR were considered as achieving good pathological response. Patients who were considered as nPR were classified into poor response group. Then, as shown in Supplementary Figure S2A, we classified the tumor stromal type into 4 categories based on the proportion of 3 components excluding residual viable tumor: inflammatory type, the proportion of inflammatory stroma exceeded that of both fibrotic stroma and necrosis by 10%; fibrotic type, the proportion of fibrotic stroma exceeded that of both inflammatory stroma and necrosis by 10%; necrotic type, the proportion of necrosis exceeded that of both inflammatory and fibrotic stroma by 10%; intermediate type, the proportion of 3 components differed from each other ≤ 10%. The evaluation of TILs and TILV referred to previous guidelines [12, 22].

### Differentially expressed genes (DEG) and immune infiltration identification by bioinformatic tools

We selected 8 fresh biopsy tissues pre-treatment (4 cases from cPR group and 4 cases from non-cPR group) from the NACT cohort to conduct RNA sequencing. The RNA sequencing was performed with the help of Beijing Genomics institution. To better identify the DEG and plot them, mRNA levels were normalized as log2(x + 1). The DEG between indicated different groups were analyzed by the helpful R package edger (lumina [23]; cluster profiler [24]) to generate a heat map. To note, the cut off for filtering for DEG was fold change > 1 and *p* < 0.05. The differentially expressed genes (DEGs) in the indicated group underwent gene ontology (GO) enrichment analysis using the R package (cluster profiler). A level of False Discovery Rates (FDR) < 0.05 was applied to determine statistical significance. Furthermore, to determine the immune cell proportions in tumor tissue samples within the indicated section, we employed the CIBERSORT algorithm [25].

### Immunohistochemistry (IHC) staining

Formalin-fixed, paraffin-embedded tumor sections were stained with IHC. Following deparaffinization with xylene and ethanol, each slide was treated with 3% hydrogen peroxide in methanol. The slides were blocked overnight with avidin-biotin at 4 °C, followed by incubation with antibodies against CD8 (ZSGB, ZA-0508), CD4 (ZSGB, ZA-0519), FOXP3 (Abcam, ab20034), PD-L1 (Dako 22C3), and ki-67 (ZSGB, ZA-0502). After washing the slides three times with PBS, biotinylated goat anti-mouse antibodies were incubated, followed by the staining of 3,3′-diaminobenzidine tetrahydro-chloride (DAB) and the counterstaining of Mayer’s hematoxylin. Two experienced pathologists observed and evaluated the staining under a microscope. Three high power fields in the peritumor stroma were randomly selected to count the density of CD8+, CD4 + and FOXP3 + T cells. PD-L1 expression was assessed using three scoring systems, including combined positive score (CPS), tumor proportion score (TPS) and immune cell proportion score (IPS), according to the PD-L1 measurement criteria [26]. CPS ≥ 1%, TPS ≥ 1% and ICS ≥ 1% were considered PD-L1 positive.

### Statistical analysis

SPSS 26.0 was used to perform statistical analyses (SPSS, Chicago, IL, USA). Clinicopathological and immune parameters were divided into high and low groups using the maximum (sensitivity + specificity) point of the Receiver Operating Characteristic (ROC) curve for the prediction of pathological response. Difference of clinicopathological characteristics and PD-L1 expression between groups were determined by Fisher’s exact test or Chi-square test. Pathological response and radiological response were correlated using Spearman’s test. Kruskal-Wallis test was used to analyze the difference of CD8+, CD4 + and FOXP3 + T cells density level between groups. Univariate and multivariate logistic regression analyses were used to analyze clinicopathological features for pathological response. The survival analysis was performed by Kaplan- Meier analysis and compared by log-rank test. The prognostic correlations were analyzed with univariate and multivariate Cox regression analyses. *P* < 0.05 was assumed statistically significant.

## Results

### Pathological response to NACT

**Fig. 2:**
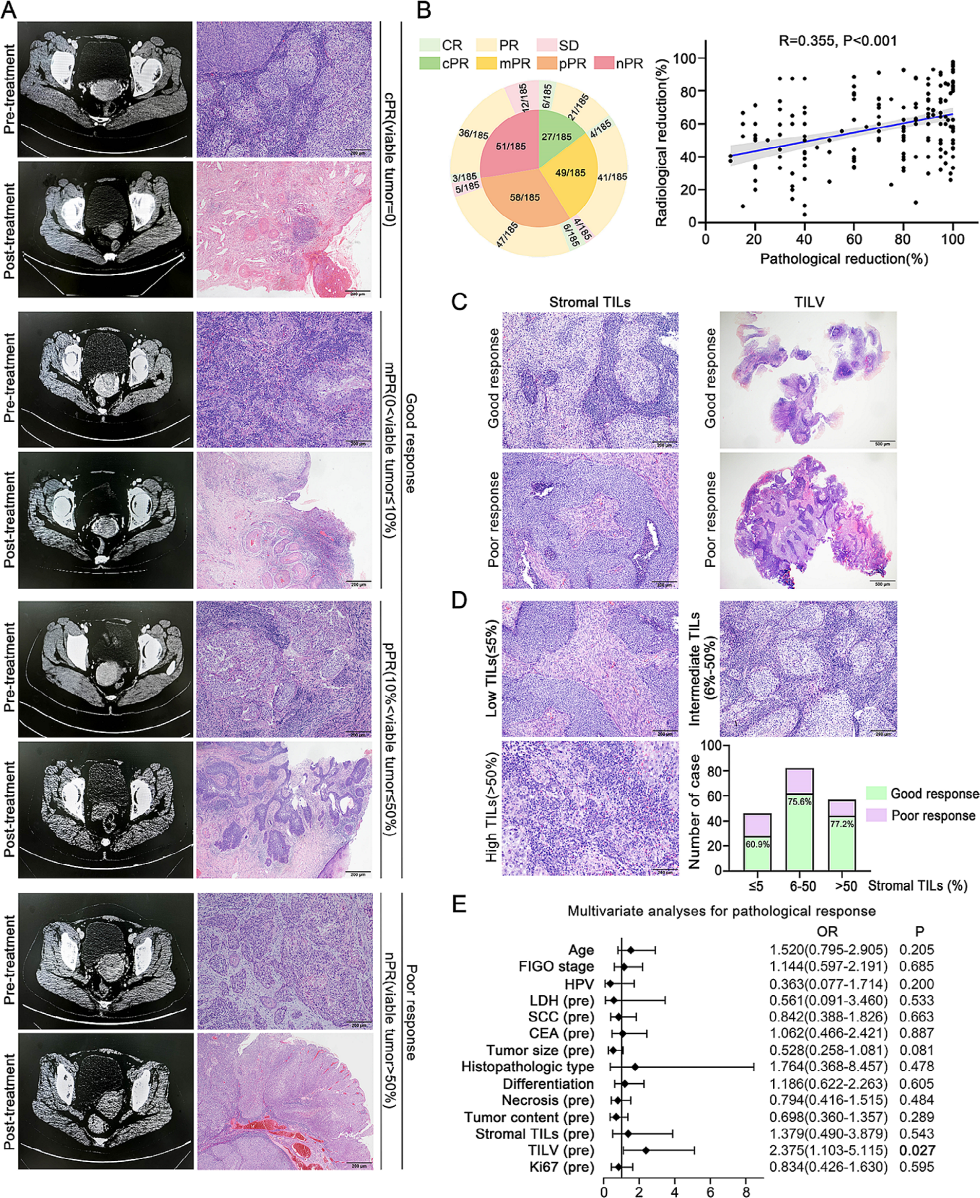
**(A)** Representative images of radiological and pathological response pre-treatment and post-treatment. **(B)** Fractions of patients with their pathological and radiological response in NACT cohort and the correlation analysis between pathological reduction and radiological reduction. **(C)** Representative images of TILs and TILV, which were associated with pathological response post-treatment. TILV (tumor-infiltrating lymphocytes volume) = stroma proportion (%) × stromal TILs proportion (%) (upper panels: stroma = 30%, stromal TILs = 80%, TILV = 2400; lower panels: stroma = 50%, stromal TILs = 5%, TILV = 250). **(D)** Representative images and fractions of various grades of stromal TILs, and the correlation with pathological response. **(E)** Multivariate analyses of clinicopathological features for pathological response are shown in forest map. LACC, locally advanced cervical cancer; cPR, complete pathological response; Proportion of residual tumor; mPR, major pathological response; pPR, partial pathological response; nPR, no pathological response; CR, complete response; PR, partial response; SD, stable disease; TILs, tumor-infiltrating lymphocytes

Clinicopathological characteristics and pathological response assessment of the NACT cohort were shown in Fig. 1C. Among the 185 total patients with LACC receiving NACT, spanning ages 19 to 67, 134 patients (72.4%, 134/185) achieved good pathological response, including 27 cPR (14.6%, 27/185), 49 mPR (26.5%, 49/185) and pPR (31.4%, 27/185). Conversely, 51 patients (31.4%, 51/185) exhibited poor pathological response. The representative CT images and H&E staining micrographs illustrating pathological response (cPR, mPR, pPR, nPR) in patients before and after NACT were displayed in Fig. 2A. The accompanying pie chart in Fig. 2B delineates the components of various pathological and radiological response in the NACT cohort. The radiographic objective response rate (CR plus PR) stood at 90.8% (168/185) post-NACT. Furthermore, statistical analysis unveiled a significant correlation between pathological and radiological reductions (*R* = 0.355, *p* < 0.001) (Fig. 2B). Among the 27 patients with cPR, 22.2% (6/27) demonstrated CR, while 77.8% (21/27) exhibited PR. These findings underscore the inadequacy of CT scans for accurately evaluating treatment effectiveness in LACC, emphasizing the imperative need to identify biomarkers capable of predicting the efficacy of NACT.

### High TILs and TILV significantly associated with good pathological response

Next, we analyzed the relationship between clinicopathological characteristics and pathological response (Table 1 and Supplementary Table 1). The investigation revealed a significant correlation between pathological response and several key factors, including lymph node metastatic (*p* = 0.005), vascular invasion (*p* = 0.017), TILs (*p* = 0.043) and TILV (*p* = 0.025) (Table 1). However, there were no statistical connections between pathological response and the remaining clinicopathological parameters, including HPV status and histologic type. Figure 2C illustrated LACC patients who exhibited good pathological response demonstrated a higher prevalence of elevated stromal TILs and TILV. In addition, we observed that a noteworthy 77.2% of patients with high grade of stromal TILs (> 50%) achieved good pathological response, surpassing those with low (≤ 5%) or intermediate (6–50%) grades of stromal TILs (Fig. 2D). Furthermore, upon multivariate analysis, high TILV emerged as an independent predictor for good pathological response (OR = 2.375, 95%CI = 1.103–5.115, *p* = 0.027) in LACC patients (Supplementary Tables 2 and Fig. 2E).

**Table 1:** Clinicopathological characteristics of the NACT cohort.

Variables	Total (N, %)	Good response (cPR/mPR/pPR) (N, %)	Poor response (nPR) (N, %)	P
**All cases**	185	134(72.4)	51(27.6)	
**Age (years)**				0.898
Median (range)	49.0(19.0–67.0)	49.0(19.0–66.0)	48.0(29.0–67.0)	
**FIGO Stage**				0.684
IB3	79(42.7)	56(41.8)	23(45.1)	
IIA2-IVA	106(57.3)	78(58.2)	28(54.9)	
**Chemotherapy regimen**				0.054
Paclitaxel + Cisplatin	87(47.0)	64(47.8)	23(45.1)	
Docetaxel + Cisplatin	70(37.8)	55(41.0)	15(29.4)	
Paclitaxel + Carboplatin	16(8.6)	10(7.5)	6(11.8)	
Others	12(6.5)	5(3.7)	7(13.7)	
**Chemotherapy cycles**				0.457
1–2	108(58.4)	76(56.7)	32(62.7)	
3	77(41.6)	58(43.3)	19(37.3)	
**Adjuvant treatment**				0.180
Yes	159(85.9)	118(88.1)	41(80.4)	
No	26(14.1)	16(11.9)	10(19.6)	
**Radiological response**				**0.001**
CR	19(10.3)	16(11.9)	3(5.9)	
PR	149(80.5)	112(83.6)	37(72.5)	
SD	17(9.2)	6(4.5)	11(21.6)	
**HPV status**				0.416
Positive	94(50.8)	66(49.3)	28(54.9)	
Negative	15(8.1)	13(9.7)	2(3.9)	
NA	76(41.1)	55(41.0)	21(41.2)	
**Tumor size (pre)(cm)**				0.078
>5.9	45(24.3)	28(20.9)	17(33.3)	
≤5.9	140(75.7)	106(79.1)	34(66.7)	
**Histologic type**				0.473
SCCA	174(94.1)	125(93.3)	49(96.1)	
AC + ASC + NEC	11(5.9)	9(6.7)	2(3.9)	
**Differentiation**				0.605
Well + Moderate	85(45.9)	60(44.8)	25(49.0)	
Poor	100(54.1)	74(55.2)	26(51.0)	
**Parametrium invasion**				0.245
Yes	9(4.9)	5(3.7)	4(7.8)	
No	176(95.1)	129(96.3)	47(92.2)	
**Lymph node metastatic**				**0.005**
Yes	37(20.0)	20(14.9)	17(33.3)	
No	148(80.0)	114(85.1)	34(66.7)	
**Vascular invasion**				**0.017**
Yes	37(20.0)	21(15.7)	16(31.4)	
No	148(80.0)	113(84.3)	35(68.6)	
**Nerve bundle invasion**				0.103
Yes	10(5.4)	5(3.7)	5(9.8)	
No	175(94.6)	129(96.3)	46(90.2)	
**LDH (pre)(u/L)**				0.617
>250.0	5(2.7)	3(2.2)	2(3.9)	
≤250.0	180(97.3)	131(97.8)	49(96.1)	
**SCC (pre)(ng/ml)**				0.662
>1.5	141(76.2)	101(75.4)	40(78.4)	
≤1.5	44(23.8)	33(24.6)	11(21.6)	
**CEA (pre)(ng/ml)**				0.946
>5.0	37(20.0)	27(20.1)	10(19.6)	
≤5.0	124(67.0)	89(66.4)	35(68.6)	
NA	24(13.0)	18(13.4)	6(11.8)	
**Necrosis (pre)**				0.483
Yes	83(44.9)	58(43.3)	25(49.0)	
No	102(55.1)	76(56.7)	26(51.0)	
**Tumor content (pre) (%)**				0.288
>85	65(35.1)	44(32.8)	21(41.2)	
≤85	120(64.9)	90(67.2)	30(58.8)	
**Stromal TILs (pre) (%)**				**0.043**
>5	139(75.1)	106(79.1)	33(64.7)	
≤5	46(24.9)	28(20.9)	18(35.3)	
**TILV (pre)**				**0.025**
>75	150(81.1)	114(85.1)	36(70.6)	
≤75	35(18.9)	20(14.9)	15(29.4)	
**Ki-67(pre) (%)**				0.595
>25	114(61.6)	81(60.4)	33(64.7)	
≤25	71(38.4)	53(39.6)	18(35.3)	

NACT, neoadjuvant chemotherapy; cPR, complete pathological response; mPR, major pathological response; pPR, partial pathological response; nPR, no pathological response; FIGO, International Federation of Gynecology and Obstetrics; HPV, human papilloma virus; NA, not available; LDH, lactate dehydrogenase; SCC, squamous cell carcinoma antigen; CEA, carcinoembryonic antigen; SCCA, squamous cell carcinoma; AC, adenocarcinoma; ASC, adenosquamous carcinoma; NEC, neuroendocrine carcinoma; TILs, tumor-infiltrating lymphocytes; TILV, tumor-infiltrating lymphocytes volume, TILV = stroma proportion (%) × stromal TILs proportion (%). Pre, pre-treatment; Post, post-treatment

### Active tumor immune microenvironment significantly correlated with good pathological response

**Fig. 3:**
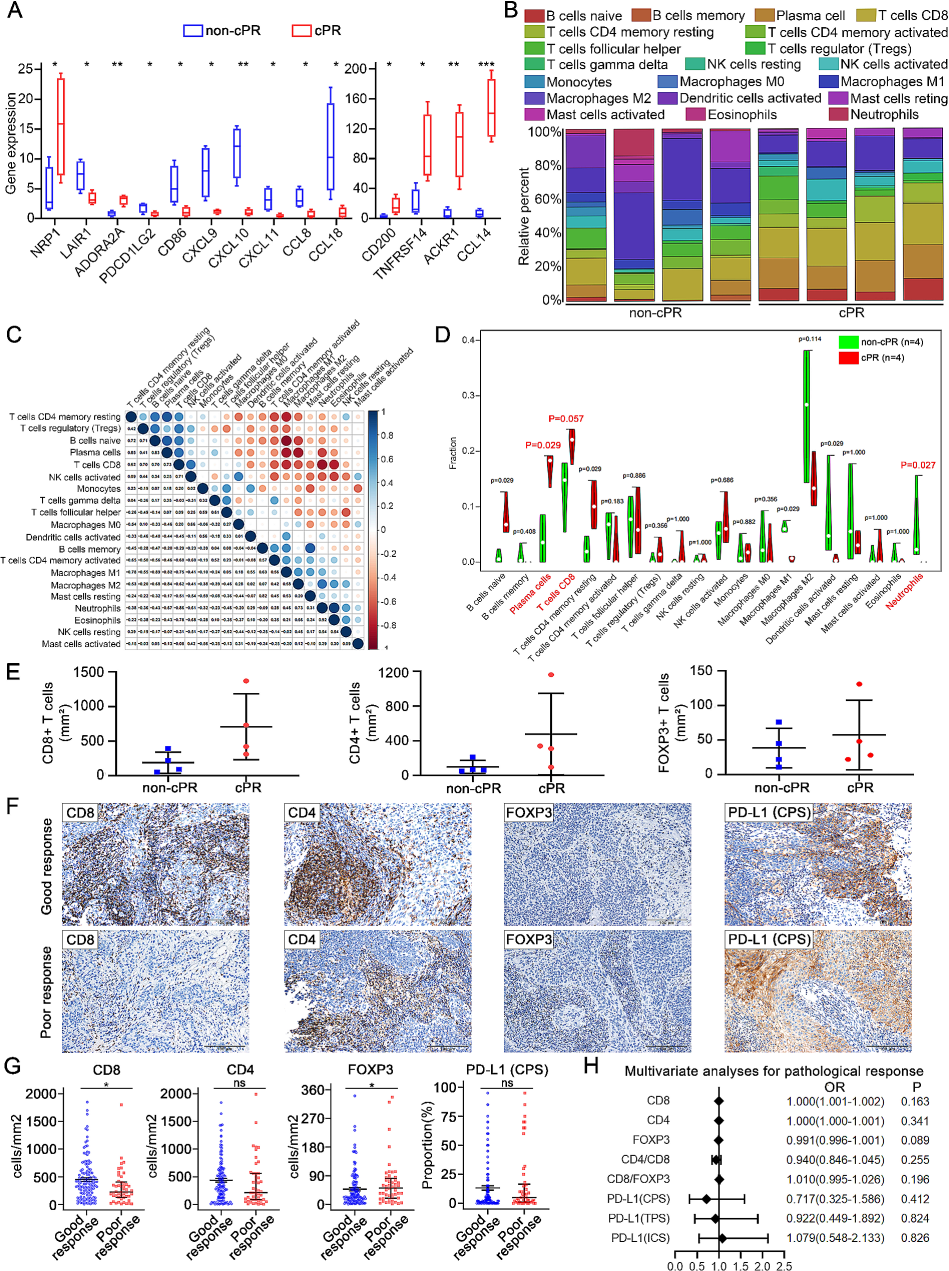
**A comparison of immune cell infiltration in 4 cPR samples and 4 non-cPR samples (The viable tumor of 4 non-cPR samples is 20%**,** 70%**,** 90% and 95%**,** respectively). (A)** Differences in chemokine and chemokine receptor expression in cPR group compared with non-cPR group. * *P* < 0.05, **P < 0.01, ***P < 0.001. **(B)** The distribution of immune cells among cPR and non-cPR samples. **(C)** Correlation among variable immune cells in cPR and non-cPR samples. **(D)** Infiltration immune cell analysis between cPR groups and non-cPR groups. **(E)** Differences of CD8, CD4 as well as FOXP3 IHC staining in cPR and non-cPR samples. **(F)** Representative images of CD8+, CD4+, FOXP3 + TILs and PD-L1 expression based on CPS pre-treatment in good response group and poor response group. **(G)** Box plots show the differences of densities of CD8+, CD4+, FOXP3 + TILs and score of PD-L1 expression based on CPS pre-treatment in good response group compared with poor response group. * *P* < 0.05; ns, no significance. **(H)** Multivariate analyses of immune markers pre-treatment for pathological response are shown in forest map. cPR, complete pathological response; PD-L1, programmed cell death ligand 1; CPS, combined positive score

To compare the tumor immune microenvironment (TIME) characteristics between pathological response of cPR and non-cPR patients, we initially examined immune genes expression in fresh tissues obtained from 4 cases each of cPR and non-cPR. Analysis using CIBERSORT and Kyoto Encyclopedia of Genes and Genomes (KEGG) clustering indicated predominant activation of the human T-cell pathway in the cPR group compared to the non-cPR group (Supplementary Figure S1A-B). Besides, the cPR group exhibited associations with some immune pathways, including positive regulation of T cell proliferation and mature B cell differentiation (Supplementary Figure S1C). Subsequent analysis focused on the expression of chemokines, chemokine receptors and immune cells in cPR and non-cPR LACC (Fig. 3A-B). According to the correlation analyses among variable immune cells, higher expression of CD8 + T cells correlated with lower expression of neutrophils (Fig. 3C). Additionally, the cPR group displayed a higher proportion of CD8 + T cells (*p* = 0.057), plasma cells (*p* = 0.029), and a lower proportion of neutrophils (*p* = 0.027) compared to the non-cPR group (Fig. 3D).

Building upon RNA sequencing analyses above, we assessed the density of CD4, CD8, Foxp3 and PD-L1-positive TILs in tumoral stromal tissues from 185 biopsy specimens. As shown in Fig. 3E, the CD8, CD4 and FOXP3 IHC staining results in the same 4 pairs of cases were consistent with the RNA sequencing data. Representative IHC staining images in good response group and poor response group were depicted in Fig. 3F. LACC cases exhibiting good pathological response demonstrated a heightened tendency density of CD8 + T cells (*p* = 0.015) and a reduced density of FOXP3 + T cells (*p* = 0.036). In addition, an elevated CD4+/CD8 + T cells ratio (*p* = 0.010) and an increased CD8+/FOXP3 + T cells ratio (*p* < 0.0001) was also observed in good response group. No significant differences were observed in CD4 + T cells density and PD-L1 expression between the two groups (Table 2; Fig. 3G). However, PD-L1 (CPS) was positively expressed in 74.6% (100/134) of samples in good response group, PD-L1 (TPS) in 70.9% (95/134) and PD-L1 (ICS) in 66.3% (89/134). Similarly, we found the 80.4% (41/51) of samples in poor response group expressed PD-L1 (CPS), 72.5% (37/51) expressed PD-L1 (TPS) and 64.7% (33/51) expressed PD-L1 (ICS). Despite conducting a multivariate logistic regression analysis on pre-treatment immune markers for pathological response, no significant results were observed (Fig. 3H). These results substantiated that elevated TILs were correlated with a favorable pathological response to NACT.

**Table 2:** Correlation of immune markers pre-treatment and pathological response.

Variables	Total	Good response (cPR, mPR, pPR) (N, %)	Poor response (nPR) (N, %)	P
**CD8 density (cells/mm²)**	185	450.3 ± 32.6	356.7 ± 65.1	**0.015**
**CD4 density (cells/mm²)**	185	436.9 ± 33.8	374.3 ± 58.8	0.151
**FOXP3 density (cells/mm²)**	185	47.7 ± 4.8	65.2 ± 10.0	**0.036**
**CD4/CD8**				**0.010**
>2.17	27(14.6)	14(10.4)	13(25.5)	
≤2.17	158(85.4)	120(89.6)	38(74.5)	
**CD8/FOXP3**				**< 0.000**
>5.24	120(64.9)	99(73.9)	21(41.2)	
≤5.24	65(35.1)	35(26.1)	30(58.8)	
**PD-L1 (CPS)**				0.410
Positive	141(76.2)	100(74.6)	41(80.4)	
Negative	44(23.8)	34(25.4)	10(19.6)	
**PD-L1 (TPS)**				0.824
Positive	132(71.4)	95(70.9)	37(72.5)	
Negative	53(28.6)	39(29.1)	14(27.5)	
**PD-L1 (IPS)**				0.826
Positive	122(65.9)	89(66.3)	33(64.7)	
Negative	63(34.1)	45(33.6)	18(35.3)	

NACT, neoadjuvant chemotherapy; cPR, complete pathological response; mPR, major pathological response; pPR, partial pathological response; nPR, no pathological response; PD-L1, programmed cell death ligand 1; CPS, combined positive score; IPS, immune cell proportion score; TPS, tumor cell proportion score

### Stromal type of post-treatment significantly correlated with LACC’s prognosis

**Fig. 4:**
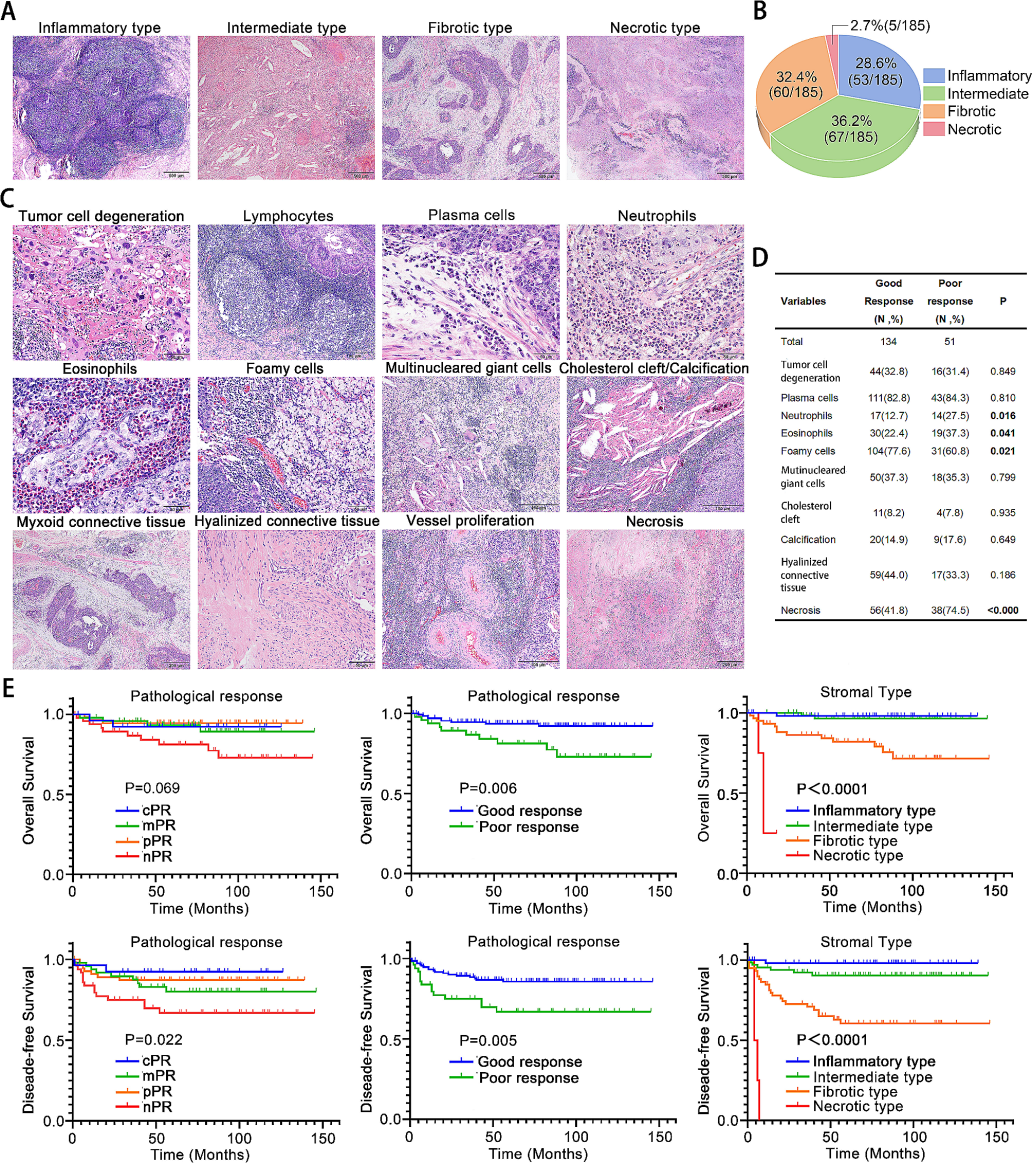
**(A)** Representative images of 4 stromal types. **(B)** Fractions of 4 stromal types. **(C)** Representative images of therapy-related histological features. Lymphocytes, plasma cells, neutrophils, eosinophils, foamy cells, multinucleared giant cells, cholesterol cleft and calcification are classified into inflammatory stroma. Loose or myxoid connective tissue, dense hyalinized connective tissue and vessel proliferation are classified into fibrotic stroma. **(D)** Correlations between therapy-related histological features and pathological response. **(E)** Kaplan-Meier survival curves for DFS and OS according to pathological response and stromal type post treatment. DFS, disease-free survival; OS, overall survival

Subsequently, our primary focus shifted to the assessment of the tumor bed stroma in resection tissues post-NACT. As illustrated in Fig. 4A-B, the 185 LACCs were subclassified into four stromal types: 53 inflammatory type, 59 fibrotic type, 5 necrotic type and 67 intermediate type. Therapeutically induced histological features were discerned in resection tissues, exemplified in Fig. 4C. Inflammatory stroma manifested as infiltration by lymphocytes, plasma cells, neutrophils, eosinophils, foamy cells, multinucleared giant cells, cholesterol clefts and calcification. Fibrotic encompassed both loose or myxoid connective tissue and dense hyalinized connective tissue. Additionally, pronounced vascularity proliferation was evident in the stroma. Noteworthy correlations were identified between pathological response and select therapy-related histological features, including neutrophils (*p* = 0.016), eosinophils (*p* = 0.041), foamy cells (*p* = 0.021) and necrosis (*p* < 0.0001) (Fig. 4D). Foamy cells were more prevalent in patients with good pathological response while neutrophils, eosinophils and necrosis were more frequent in the poor response group. Multivariate analysis further identified foamy cells (HR = 0.409, 95%CI = 0.207–0.808, *p* = 0.010) and hyalinized connective tissue (HR = 2.195, 95%CI = 1.110–4.341, *p* = 0.024) as independent factors for DFS (Supplementary Figure S2B).

Subsequent Kaplan-Meier analysis was conducted to assess the impact of pathological response and stromal type post-NACT on the prognosis of LACC patients (Fig. 4E). Good pathological response correlated with better OS (*p* = 0.006) and DFS (*p* = 0.005). Concerning stromal type, patients with inflammatory type and intermediate type exhibited significantly longer OS and DFS, but there was no significant difference between them. Remarkably, the fibrotic type displayed an intermediate prognosis, while necrotic type had the poorest prognosis for both DFS and OS (both *p* < 0.0001). Crucially, multivariate analysis established stromal type as an independent prognostic factor for both OS (HR = 3.749, 95%CI = 1.861–7.552, *p* = 0.004) and DFS (HR = 4.719, 95%CI = 2.634–8.456, *p* < 0.0001) in LACC (Table 3).

**Table 3:** Univariate and multivariate analyses for DFS and OS.

Variables	Univariate analyses		Multivariate analyses	
	HR (95%CI)	P	HR (95%CI)	P
OS				
**Age** (> 48 yeas vs. ≤48 years)	5.127(1.502–17.508)	**0.009**	5.804(1.682–20.030)	**0.005**
**FIGO Stage** (IB3 vs. IIA2-IVA)	2.405(0.874–6.623)	0.089		
**HPV** (Positive vs. Negative)	1.377(0.663–2.859)	0.391		
**Tumor size (pre)** (> 5.9cm vs. ≤5.9cm)	0.911(0.304–2.728)	0.868		
**Histologic type** (SCCA vs. others)	0.830(0.111–6.211)	0.856		
**Differentiation** (Well + Moderate vs. Poor)	0.812(0.338–1.954)	0.642		
**Stromal invasion level** (< 1/3 vs. 1/3 − 2/3 vs.>2/3)	2.500(1.377–4.540)	**0.003**		
**Parametrium invasion** (Yes vs. No)	6.735(2.233–20.310)	**0.001**	4.903(1.347–17.851)	**0.016**
**Lymph node metastatic** (Yes vs. No)	6.035(2.494–14.602)	**< 0.000**		
**Vascular invasion** (Yes vs. No)	7.123(2.905–17.467)	**< 0.000**	5.764(2.185–15.206)	**< 0.000**
**Nerve bundle invasion** (Yes vs. No)	2.490(0.575–10.778)	0.222		
**Pathological response** (Good response vs. Poor response)	0.341(0.142–0.820)	**0.016**		
**Stromal type** (Inflammatory vs.Intermediate vs.Fibrotic vs.Necrotic)	6.353(2.867–14.081)	**< 0.000**	3.749(1.861–7.552)	**0.004**
**DFS**				
**Age** (> 48 yeas vs. ≤ 48 years)	1.406(0.704–2.808)	0.334		
**FIGO Stage** (IB3 vs. IIA2-IVA)	1.430(0.708–2.890)	0.319		
**HPV status** (Positive vs. Negative)	2.514(0.330–19.120)	0.373		
**Tumor size** (pre) (> 5.9cm vs. ≤5.9cm)	0.692(0.287–1.666)	0.411		
**Histologic type** (SCCA vs. others)	0.930(0.223–3.876)	0.921		
**Differentiation** (Well + Moderate vs. Poor)	0.694(0.357–1.350)	0.282		
**Stromal invasion level** (< 1/3 vs. 1/3 − 2/3 vs.>2/3)	2.233(1.451–3.437)	**< 0.000**		
**Parametrium invasion** (Yes vs. No)	3.426(1.205–9.739)	**0.021**		
**Lymph node metastatic** (Yes vs. No)	6.097(3.096–12.008)	**< 0.000**		
**Vascular invasion** (Yes vs. No)	5.973(3.037–11.749)	**< 0.000**	4.214(2.098–8.467)	**< 0.000**
**Nerve bundle invasion** (Yes vs. No)	5.727(2.340-14.018)	**< 0.000**		
**Pathological response** (Good response vs. Poor response)	0.414(0.210–0.815)	**0.011**		
**Stromal type** (Inflammatory vs.Intermediate vs.Fibrotic vs.Necrotic)	5.696(3.101–10.464)	**< 0.000**	4.719(2.634–8.456)	**< 0.000**

HR, hazard ratio; OS, overall survival; DFS, disease-free survival; FIGO, International Federation of Gynecology and Obstetrics; HPV, human papilloma virus; NACT, neoadjuvant chemotherapy; SCCA, squamous cell carcinoma

### Association of TIME with pathological response to NACIT in LACC

To enhance our understanding of the evaluation of pathological response post-NACT in LACC patients, we extended our examination to the NACIT cohort. Baseline characteristics of the 7 patients undergoing NACIT are detailed in Supplementary Tables 3 and Supplementary Figure S3A, encompassing individuals aged 26 to 66, with follow-up periods ranging from 1 to 15 months. Notably, 85.7% (6/7) of the cohort exhibited good pathological response, comprising 1 case of cPR, 4 cases of mPR and 1 case of pPR. It is noteworthy that the residual viable tumor in all 6 good response cases were all ≤ 15%. Only one case was assigned to nPR, with a residual viable tumor of 65%. Importantly, no recurrence or mortality was observed in any of the 7 cases. The pPR case revealed lymph node metastatic and nerve bundle invasion with a residual viable tumor of 15%. Similarly, the nPR case exhibited lymph node metastatic and vascular invasion. Conversely, the remaining 5 cases displayed neither lymph node metastatic nor vascular or nerve bundle invasion. Representative H&E stain and IHC images were presented in Supplementary Figure S3B. PD-L1 (CPS) positive was observed in all cases, particularly with relatively high expression in cPR and mPR cases. Furthermore, 87.5% (5/6) of the good response cases presented abundant stromal TILs pre-treatment, including CD8 + T cells and CD4 + T cells.

## Discussion

To date, a standard pathological evaluation system for cervical cancer remains elusive. While there is a general consensus to defining cPR, the categorization of non-complete response varies across studies [19]. Previous studies have typically classified pathological response into different grades based on the residual tumor size or invasive depth [19]. In our approach, we quantified the percentage of residual viable tumor in the cervical primary region, drawing inspiration from definitions in lung cancer (mPR) [27] and melanoma (pPR and nPR) [21]. Consequently, we devised a four-tiered pathological response evaluation system for cervical cancer post-NACT, encompassing cPR, mPR, pPR and nPR. This system, not reliant on tumor size or invasive depth, offers a more nuanced reflection of tumor overall state and treatment response. Importantly, our pathological system exhibited a robust correlation with the RECIST in radiological response assessment. Pathological assessment can additionally reveal inflammatory or fibrotic stroma that are challenging to discern through CT scans. The synergistic application of both CT imaging and pathological assessment may improve the precision in evaluating the effectiveness of neoadjuvant therapy.

The immune microenvironment emerges as intricately linked to the pathological response to NACT [28]. Stromal TILs have been recognized as a prognostic indicator and a predictor of pathological response post-NACT, playing a pivotal role in tumor immunity [11, 29, 30]. Aligning with prior studies, we found that heightened expression levels of stromal TILs correlated with a more favorable pathological response. Our study also identified the T cell proliferation pathway, particularly evaluated expression of CD8 + T cells, as crucial for achieving cPR. This finding was subsequently validated through IHC staining of 185 LACC biopsy specimens. The increased presence of CD8 + T cells in biopsies appears indicative of a more favorable pathological response post-NACT. Tumor infiltrating CD8 + T cells are widely recognized as pivotal guardians against tumorigenesis, with their ability to mount a robust anti-tumor response. Conversely, FOXP3 + T cells play a regulatory role by impeding the proliferation and activation of CD8 + T cells, as evidenced by pertinent studies [9]. An elevated CD8/FOXP3 ratio emerges as a notable prognostic indicator for enhanced survival across diverse cancer types, including rectal cancer, esophageal squamous cell cancer, lung adenocarcinoma and cervical cancer [9, 31–33]. It may be the balance or interaction between CD8 + and FOXP3 + T cells in the tumor microenvironment rather than the quantity of TILs alone, determines clinical outcomes. In this study, we found that a high infiltration of CD8 + T cells coupled with low levels of FOXP3 + T cells in the tumor stroma may aid in selecting patients who are clinically responsive to chemotherapy, undergoing the influence of host pretreatment immune status on chemotherapy effectiveness. Additionally, in the NACIT cohort, 6 out of 7 LACC patients exhibited a favorable pathological response alongside a high level of immune cell infiltration or positive PD-L1 expression in biopsy samples. This may indicate the great potential of immunotherapy in LACC. Indeed, the co-expression of PD-L1 and TILs have been reported in various cancers [10, 34]. When PD-L1 on tumor tissue binds to its ligand PD-1, it triggers an immunosuppressive signal, hindering immune cells to attack tumor. Elevated levels of TILs suggest an activated immune environment within the body. When alleviating PD-L1 inhibition on TILs, more immune cells can be activated to identify and eliminate tumor cells [35, 36]. Hence, the utilization of immune checkpoint inhibitors, such as PD-L1/PD-1 blockers, may yield a potent and efficacious response in cases where PD-L1 and TILs are co-expressed. Nevertheless, the potential of PD-L1 and TILs as biomarkers for predicting treatment response and prognosis in cervical cancer requires further exploration.

Currently, studies on cervical cancer post-NACT predominantly focus on tumor staging, histological types and conventional pathological risk factors like lymphatic vascular invasion [28, 37]. However, the morphological alterations of tumor cells and stroma and their impact on prognosis remain underexplored. Our study directs attention to the stroma, encompassing inflammation, fibrosis, and necrosis. Tumor-associated inflammatory infiltration has long been recognized as a host response and a crucial factor in anti-tumor actively. Notably, good responders exhibit dense TILs and plasma cells in post-treatment excised specimens, pivotal for bolstering the anti-tumor response [17]. Other immune cell subsets, including Tregs and M2 macrophages, have also been implicated in promoting tissue repair, suggesting a complex interplay between specific immune cell subsets, tumor cell death and tissue repair [38–40]. Our findings suggest that a broader inflammation in the tumor bed may correlate with a more favorable prognostic assessment, warranting further exploration of treatment options aimed at enhancing the host immune response in patients. In addition to inflammatory infiltration, fibrosis and necrosis represent alternative forms of tumor regression. Some studies have explored these aspects in soft tissue tumors, with necrosis post-NACT considered an adverse prognostic factor, while tumor fibrosis seems to reflect a reparative response to initial tumor necrosis [41, 42]. The collagen fibers, serve as the primary components of the extracellular matrix, origin from cancer-associated fibroblasts (CAFs). Chemotherapy may activate CAFs and prompt the accumulation of collagen fibers within the stroma. This may facilitate the formation of a natural barrier, leading to immune resistance and influencing subsequent anti-tumor therapies. Consequently, it may contribute to a poor prognosis of patients [43, 44]. Our study underscores that the stromal type post-treatment emerges as an independent prognostic factor in LACC patients, which may offer guidance for prognosis and clinical decisions. However, the actual prognostic impact may hinge on the interplay of multiple factors. It is not advisable to rely solely on this factor for making medical decision. Rather, it should be assessed in conjunction with other pertinent clinical parameters.

Despite developing a straightforward and prognostically significant system for grading the response of LACC patients to NACT, our study has limitations. The retrospective nature of our pathological response evaluation system calls for more robust evidence through a meticulous and randomized prospective clinical study. Moreover, as all specimens originated from a single center, potential selection bias arises, necessitating further validated of our results in multi-center studies with a larger sample size.

### Electronic supplementary material

Below is the link to the electronic supplementary material.

Supplementary Material 1

## Data Availability

The datasets used or analyzed during the current study are available from the corresponding author on reasonable request.

## Abbreviations

CCRT: Concurrent chemoradiotherapy
LACC: Locally advanced cervical cancer
NACT: Neoadjuvant chemotherapy
TILs: Tumor-infiltrating lymphocytes
TILV: Tumor infiltrating lymphocytes volume
SYSUCC: Sun Yat-sen University Cancer Center
FIGO: International Federation of Gynecology and Obstetrics
CT: Pelvic computed tomography
PD: Progressive disease
SD: Stable disease
PR: Partial response
CR: Tumor complete resolution
cPR: Complete pathological response
mPR: Major pathological response
pPR: Partial pathological response
nPR: No pathological response
DEG: Differentially expressed genes
GO: Gene ontology
FDR: False Discovery Rates
HE: Hematoxylin-eosin staining
IHC: Immunohistochemistry
FOXP3: Forkhead box protein P3
DAB: Diaminobenzidine tetrahydro-chloride
PD-L1: Programmed death-ligand 1
CPS: Combined positive score
TPS: Tumor proportion score
IPS: Immune cell proportion score
ROC: Receiver Operating Characteristic
TIME: Tumor immune microenvironment
KEGG: Kyoto Encyclopedia of Genes and Genomes
HPV: Human papilloma virus
NA: Not available
LDH: Lactate dehydrogenase
SCC: Squamous cell carcinoma antigen
CEA: Carcinoembryonic antigen
SCCA: Squamous cell carcinoma
AC: Adenocarcinoma
ASC: Adenosquamous carcinoma
NEC: Neuroendocrine carcinoma
LNM: Lymph node metastatic
HR: Hazard ratio
OS: Overall survival
DFS: Disease-free survival
OR: Odds ratio
NACIT: Neoadjuvant chemotherapy combined with immunotherapy
