# Supplementary material for: Evaluation of pathological response to neoadjuvant chemotherapy in locally advanced cervical cancer
Source: J Transl Med. 2024 Jul 14;22:655. doi: 10.1186/s12967-024-05482-3 (PMC11247755; doi:10.1186/s12967-024-05482-3)
Supplement: Supplementary file 1 — Supplementary Material 1 [file 12967_2024_5482_MOESM1_ESM.docx]

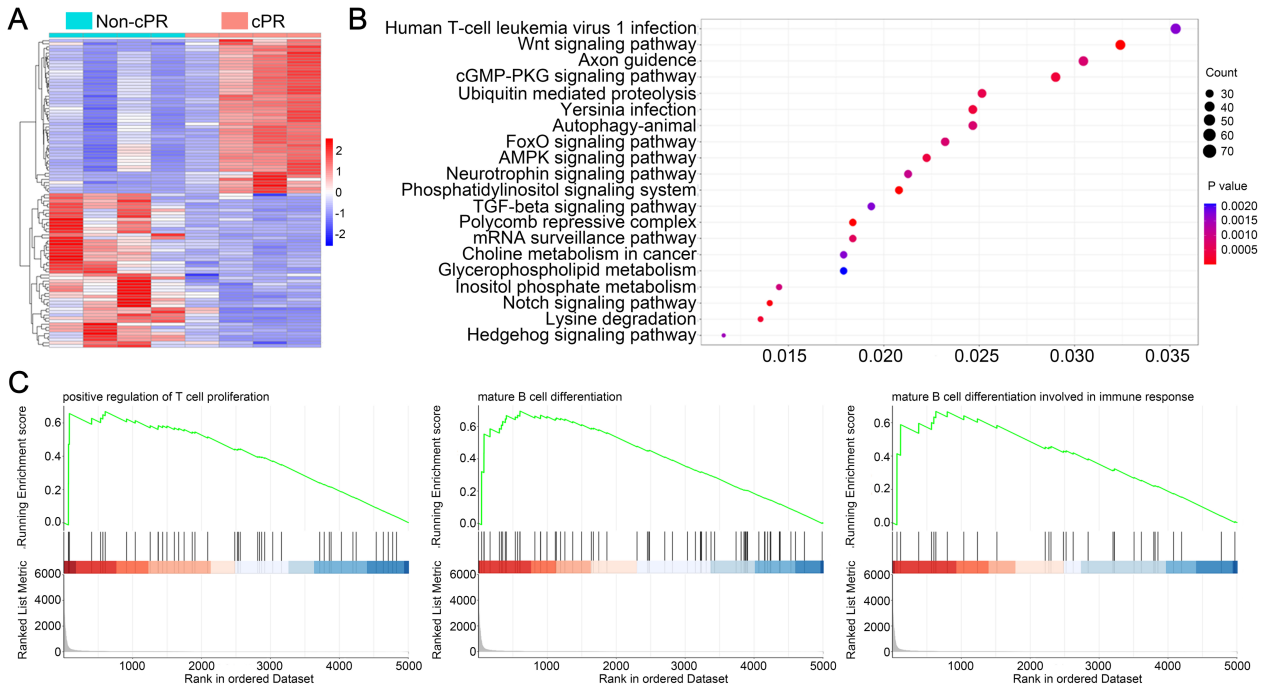


**Supplementary Figure S1 (A)** Heat map describing genes up or down regulated in cPR group compared to non-cPR group. **(B)** KEGG analysis enriched pathway changed in cPR group compared with non-cPR group. **(C)** Immune pathways that associated with cPR.

cPR, complete pathologic response; KEGG, Kyoto Encyclopedia of Genes and Genomes.


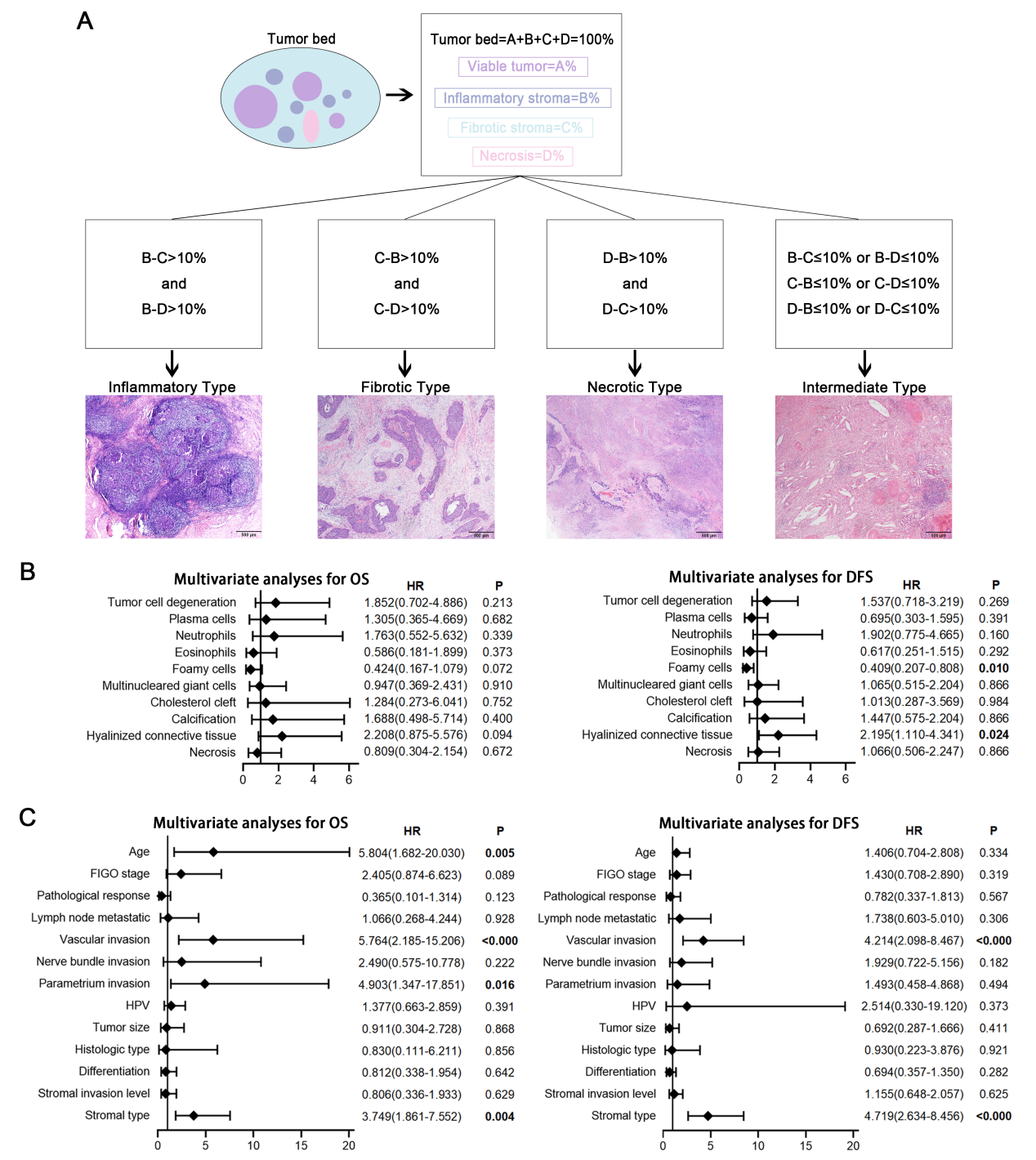


**Supplementary Figure S2 (A)** Classification of 4 stromal types after NACT. **(B)** Multivariate analyses of therapy-related histological features for OS and DFS are shown in forest map. **(C)** Multivariate analyses of clinicopathological characteristics for OS and DFS are shown in forest map.

NACT, neoadjuvant chemotherapy; DFS, disease-free survival; OS, overall survival.


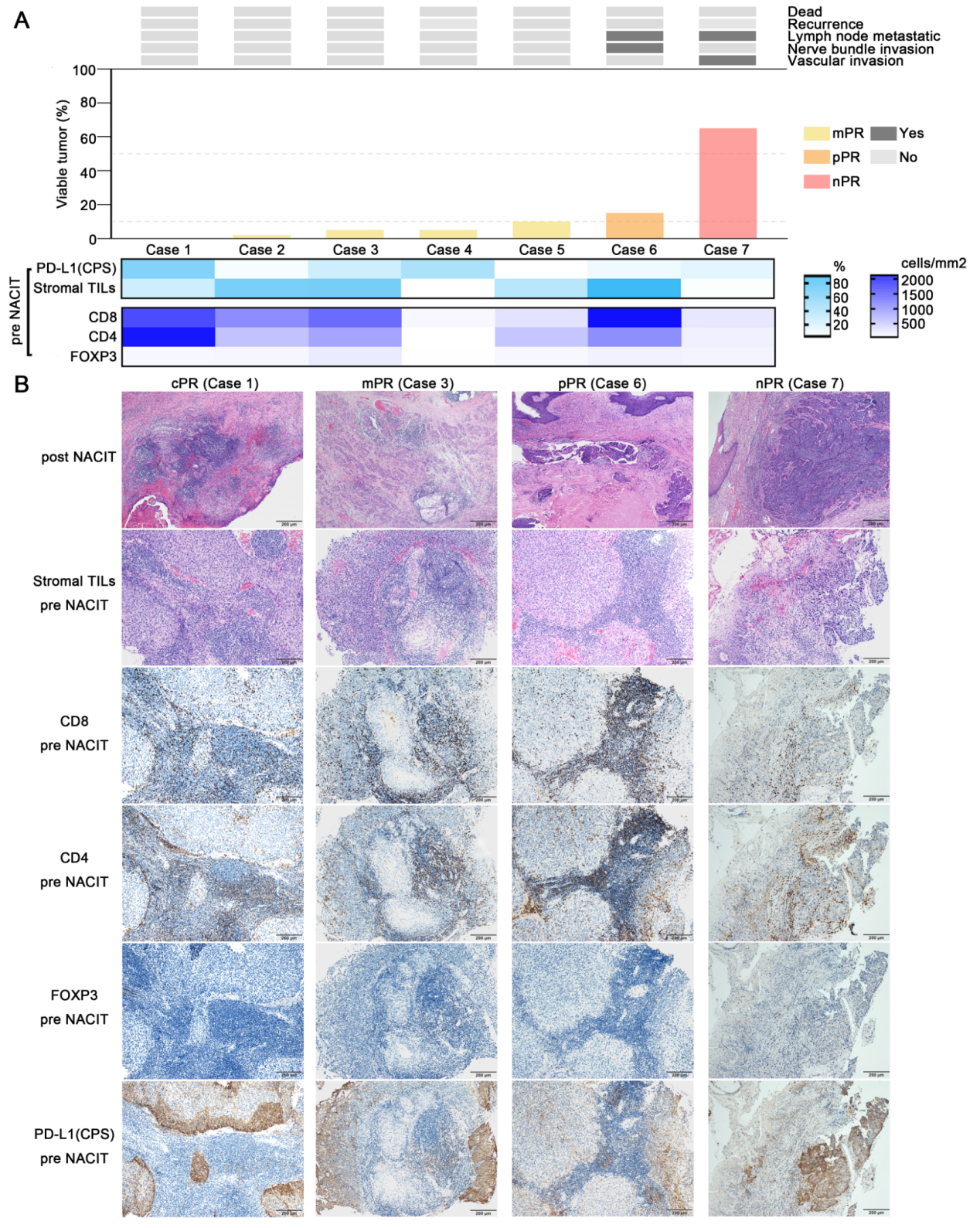


**Supplementary Figure S3 (A)** Clinicopathological and immune infiltration characteristics of 7 NACIT samples. **(B)** Representative HE and IHC images of variable pathological response in NACIT samples.

NACIT, neoadjuvant chemotherapy combined with immunotherapy; HE, hematoxylin-eosin staining; IHC, immunohistochemistry.

**Supplementary Table 1. Clinicopathological characteristics and immune markers of 4 pathological responses in NACT cohort.**

| **Variables** | **Total (N, %)** | **Good response**  **(N=134)** | | | **Poor response**  **(N=51)** | **P** |
| --- | --- | --- | --- | --- | --- | --- |
|  |  | **cPR(N, %)** | **mPR(N, %)** | **pPR(N, %)** | **nPR(N, %)** |  |
| **All cases** | 185 | 27(14.6) | 49(26.5) | 58(31.3) | 51(27.6) |  |
| **Age (years)** |  |  |  |  |  | 0.998 |
| Median (range) | 49(19-67) | 49(31-59) | 49(32-66) | 49.5(19-65) | 48(29-67) |  |
| **FIGO Stage** |  |  |  |  |  | 0.769 |
| IB3 | 79(42.7) | 10(37.0) | 19(38.8) | 27(46.6) | 23(45.1) |  |
| IIA2-IVA | 106(57.3) | 17(63.0) | 30(61.2) | 31(53.4) | 28(54.9) |  |
| **Chemotherapy regimen** |  |  |  |  |  | 0.397 |
| Paclitaxel+Cisplatin | 87(47.0) | 11(40.7) | 25(51.0) | 28(48.3) | 23(45.1) |  |
| Docetaxel+Cisplatin | 70(37.8) | 14(51.9) | 18(36.7) | 23(39.7) | 15(29.4) |  |
| Paclitaxel+Carboplatin | 16(8.6) | 2(7.4) | 3(6.1) | 5(8.6) | 6(11.8) |  |
| Others | 12(6.5) | 0 | 3(6.1) | 2(3.4) | 7(13.7) |  |
| **Chemotherapy cycles** |  |  |  |  |  | 0.903 |
| 1-2 | 108(58.4) | 15(55.6) | 28(57.1) | 33(56.9) | 32(62.7) |  |
| 3 | 77(41.6) | 12(44.4) | 21(42.9) | 25(43.1) | 19(37.3) |  |
| **Adjuvant treatment** |  |  |  |  |  | 0.242 |
| Yes | 159(85.9) | 25(92.6) | 45(91.8) | 48(82.8) | 41(80.4) |  |
| No | 26(14.1) | 2(7.4) | 4(8.2) | 10(17.2) | 10(19.6) |  |
| **Radiological response** |  |  |  |  |  | **0.013** |
| CR | 19(10.3) | 6(22.2) | 4(8.2) | 6(10.3) | 3(5.9) |  |
| PR | 149(80.5) | 21(77.8) | 42(85.7) | 49(84.5) | 37(72.5) |  |
| SD | 17(9.2) | 0 | 3(6.1) | 3(5.2) | 11(21.6) |  |
| **HPV status** |  |  |  |  |  | 0.796 |
| Positive | 94(50.8) | 12(44.4) | 24(49.0) | 30(51.7) | 28(54.9) |  |
| Negative | 15(8.1) | 4(14.8) | 4(8.2) | 5(8.6) | 2(3.9) |  |
| NA | 76(41.1) | 11(40.7) | 21(42.9) | 23(39.7) | 21(41.2) |  |
| **Tumor size (pre)(cm)** |  |  |  |  |  | 0.346 |
| >5.9 | 45(24.3) | 6(22.2) | 11(22.4) | 11(19.0) | 17(33.3) |  |
| ≤5.9 | 140(75.7) | 21(77.8) | 38(77.6) | 47(81.0) | 34(66.7) |  |
| **Histologic type** |  |  |  |  |  | 0.877 |
| SCCA | 174(94.1) | 25(92.6) | 46(93.9) | 54(93.1) | 49(96.1) |  |
| Others | 11(5.9) | 2(7.4) | 3(6.1) | 4(6.9) | 2(3.9) |  |
| **Differentiation** |  |  |  |  |  | 0.303 |
| Well + Moderate | 85(45.9) | 11(40.7) | 27(55.1) | 22(37.9) | 25(49.0) |  |
| Poor | 100(54.1) | 16(59.3) | 22(44.9) | 36(62.1) | 26(51.0) |  |
| **Parametrium invasion** |  |  |  |  |  | 0.345 |
| Yes | 9(4.9) | 0 | 1(2.0) | 4(6.9) | 4(7.8) |  |
| No | 176(95.1) | 27(100.0) | 48(98.0) | 54(93.1) | 47(92.2) |  |
| **Lymph node metastatic** |  |  |  |  |  | **0.015** |
| Yes | 37(20.0) | 1(3.7) | 9(18.4) | 10(17.2) | 17(33.3) |  |
| No | 148(80.0) | 26(96.3) | 40(81.6) | 48(82.8) | 34(66.7) |  |
| **Vascular invasion** |  |  |  |  |  | **0.009** |
| Yes | 37(20.0) | 0 | 8(16.3) | 13(22.4) | 16(31.4) |  |
| No | 148(80.0) | 27(100.0) | 41(83.7) | 45(77.6) | 35(68.6) |  |
| **Nerve bundle invasion** |  |  |  |  |  | 0.207 |
| Yes | 10(5.4) | 0 | 1(2.0) | 4(6.9) | 5(9.8) |  |
| No | 175(94.6) | 27(100.0) | 48(98.0) | 54(93.1) | 46(90.2) |  |
| **LDH (pre)(u/L)** |  |  |  |  |  | 0.935 |
| >250.0 | 5(2.7) | 0 | 1(2.0) | 2(3.4) | 2(3.9) |  |
| ≤250.0 | 180(97.3) | 27(100.0) | 48(98.0) | 56(96.6) | 49(96.1) |  |
| **SCC (pre)(ng/ml)** |  |  |  |  |  | 0.974 |
| >1.5 | 141(76.2) | 20(74.1) | 37(75.5) | 44(75.9) | 40(78.4) |  |
| ≤1.5 | 44(23.8) | 7(25.9) | 12(24.5) | 14(24.1) | 11(21.6) |  |
| **CEA (pre)(ng/ml)** |  |  |  |  |  | 0.619 |
| >5.0 | 37(20.0) | 4(14.8) | 11(22.4) | 12(20.7) | 10(19.6) |  |
| ≤5.0 | 124(67.0) | 22(81.5) | 31(63.3) | 36(62.1) | 35(68.6) |  |
| NA | 24(13.0) | 1(3.7) | 7(14.3) | 10(17.2) | 6(11.8) |  |
| **Necrosis (pre)** |  |  |  |  |  | 0.877 |
| Yes | 83(44.9) | 12(44.4) | 20(40.8) | 26(44.8) | 25(49.0) |  |
| No | 102(55.1) | 15(55.6) | 29(59.2) | 32(55.2) | 26(51.0) |  |
| **Tumor content (pre) (%)** |  |  |  |  |  | 0.145 |
| >85 | 65(35.1) | 13(48.1) | 12(24.5) | 19(32.8) | 21(41.2) |  |
| ≤85 | 120(64.9) | 14(51.9) | 37(75.5) | 39(67.2) | 30(58.8) |  |
| **Stromal TILs (pre) (%)** |  |  |  |  |  | 0.174 |
| >5 | 139(75.1) | 23(85.2) | 39(79.6) | 44(75.9) | 33(64.7) |  |
| ≤5 | 46(24.9) | 4(14.8) | 10(20.4) | 14(35.3) | 18(35.3) |  |
| **TILV (pre)** |  |  |  |  |  | 0.137 |
| >75 | 150(81.1) | 24(88.9) | 42(85.7) | 48(82.8) | 36(70.6) |  |
| ≤75 | 35(18.9) | 3(11.1) | 7(14.3) | 10(17.2) | 15(29.4) |  |
| **Ki-67(pre) (%)** |  |  |  |  |  | 0.943 |
| >25 | 114(61.6) | 17(63.0) | 29(59.2) | 35(60.3) | 33(64.7) |  |
| ≤25 | 71(38.4) | 10(37.0) | 20(40.8) | 23(39.7) | 18(35.3) |  |
| **CD8 density (cells/mm²)** | 185 | 494.2±94.1 | 427.5±49.2 | 450.5±46.8 | 356.7±65.1 | 0.114 |
| **CD4 density (cells/mm²)** | 185 | 474.0±91.0 | 437.5±57.0 | 420.2±46.0 | 374.3±58.8 | 0.554 |
| **FOXP3 density (cells/mm²)** | 185 | 48.8±12.0 | 40.6±5.8 | 53.1±8.4 | 65.2±10.0 | 0.147 |
| **CD4/CD8** |  |  |  |  |  | 0.071 |
| >2.17 | 27(14.6) | 2(7.4) | 5(10.2) | 7(12.1) | 13(25.5) |  |
| ≤2.17 | 158(85.4) | 25(92.6) | 44(89.8) | 51(87.9) | 38(74.5) |  |
| **CD8/FOXP3** |  |  |  |  |  | **<0.000** |
| >5.24 | 120(64.9) | 20(74.1) | 38(77.6) | 41(70.7) | 21(41.2) |  |
| ≤5.24 | 65(35.1) | 7(25.9) | 11(22.4) | 17(29.3) | 30(58.8) |  |
| **PD-L1 (CPS)** |  |  |  |  |  | 0.330 |
| Positive | 141(76.2) | 17(63.0) | 37(75.5) | 46(79.3) | 41(80.4) |  |
| Negative | 44(23.8) | 10(37.0) | 12(24.5) | 12(20.7) | 10(19.6) |  |
| **PD-L1 (TPS)** |  |  |  |  |  | 0.467 |
| Positive | 132(71.4) | 16(59.3) | 35(71.4) | 44(75.9) | 37(72.5) |  |
| Negative | 53(28.6) | 11(40.7) | 14(28.6) | 14(24.1) | 14(27.5) |  |
| PD-L1 (ICS) |  |  |  |  |  | 0.781 |
| Positive | 122(65.9) | 18(66.7) | 35(71.4) | 36(62.1) | 33(64.7) |  |
| Negative | 63(34.1) | 9(33.3) | 14(28.6) | 22(37.9) | 18(35.3) |  |

NACT, neoadjuvant chemotherapy; cPR, complete pathological response; mPR, major pathological response; pPR, partial pathological response; nPR, no pathological response; FIGO, International Federation of Gynecology and Obstetrics; HPV, human papilloma virus; NA, not available; LDH, lactate dehydrogenase; SCC, squamous cell carcinoma antigen; CEA, carcinoembryonic antigen; SCCA, squamous cell carcinoma; TILs, tumor-infiltrating lymphocytes; TILV, tumor-infiltrating lymphocytes volume, TILV= stroma proportion (%) × stromal TILs proportion (%). Pre, pre-treatment; Post, post-treatment; PD-L1, programmed cell death ligand 1; CPS, combined positive score; IPS, immune cell proportion score; TPS, tumor cell proportion score.

**Supplementary Table 2. Univariate and multivariate analyses for pathological response.**

| **Variables** | **Univariate analyses** | | **Multivariate analyses** | |
| --- | --- | --- | --- | --- |
|  |  |  |  |  |
|  | **OR (95%CI)** | **P** | **OR (95%CI)** | **P** |
| **Age (>48 yeas vs. ≤48 years)** | 1.520(0.795-2.905) | 0.205 |  |  |
| **FIGO Stage (IB3 vs. IIA2-IVA)** | 1.144(0.597-2.191) | 0.685 |  |  |
| **HPV (Positive vs. Negative)** | 0.363(0.077-1.714) | 0.200 |  |  |
| **LDH (pre) (>250.0u/L vs.≤250.0u/L)** | 0.561(0.091-3.460) | 0.533 |  |  |
| **SCC (pre) (>1.5ng/mL vs.≤1.5ng/ml)** | 0.842(0.388-1.826) | 0.663 |  |  |
| **CEA (pre) (>5.0ng/mL vs.≤5.0ng/ml)** | 1.062(0.466-2.421) | 0.887 |  |  |
| **Tumor size (pre) (>5.9cm vs.≤5.9cm)** | 0.528(0.258-1.081) | 0.081 |  |  |
| **Histologic type (SCCA vs. others)** | 1.764(0.368-8.457) | 0.478 |  |  |
| **Differentiation (Well+Moderate vs. Poor)** | 1.186(0.622-2.263) | 0.605 |  |  |
| **Necrosis (pre) (Yes vs. No)** | 0.794(0.416-1.515) | 0.484 |  |  |
| **Tumor content (pre) (>85% vs.≤85%)** | 0.698(0.360-1.357) | 0.289 |  |  |
| **Stromal TILs (pre) (>5% vs.≤5%)** | 2.065(1.016-4.198) | **0.045** |  |  |
| **TILV (pre) (>75 vs.≤75)** | 2.375(1.103-5.115) | **0.027** | 2.375(1.103-5.115) | **0.027** |
| **Ki-67 (pre) (>25% vs.≤25%)** | 0.834(0.426-1.630) | 0.595 |  |  |

OR, odds ratio; FIGO, International Federation of Gynecology and Obstetrics; HPV, human papilloma virus; LDH, lactate dehydrogenase; SCC, squamous cell carcinoma antigen; CEA, carcinoembryonic antigen; SCCA, squamous cell carcinoma; TILs, tumor-infiltrating lymphocytes; TILV, tumor-infiltrating lymphocytes volume, TILV= stroma proportion (%) × stromal TILs proportion (%); Pre, pre-treatment.

**Supplementary Table 3. Baseline characteristics of patients receive NACIT (n=7).**

| **Characteristics** | **N (%)** |
| --- | --- |
| **Age(years)(median,range)** | 51(26-66) |
| **Follow-up months (Average, range)** | 5(1-15) |
| **FIGO Stage** |  |
| IB3 | 1(14.3) |
| IIA2 | 3(42.9) |
| IIB | 1(14.3) |
| IIIC1 | 2(28.6) |
| **Histologic type** |  |
| Squamous cell carcinoma | 7(100.0) |
| **Differentiation** |  |
| Moderate | 4(57.1) |
| Poor | 3(42.9) |

NACIT, neoadjuvant chemotherapy combined with immunotherapy.
